# Synthesis, characterization, and cytotoxicity in human erythrocytes of multifunctional, magnetic, and luminescent nanocrystalline rare earth fluorides

**DOI:** 10.1007/s11051-015-3191-2

**Published:** 2015-10-05

**Authors:** Tomasz Grzyb, Lucyna Mrówczyńska, Agata Szczeszak, Zbigniew Śniadecki, Marcin Runowski, Bogdan Idzikowski, Stefan Lis

**Affiliations:** Department of Rare Earths, Faculty of Chemistry, Adam Mickiewicz University, Umultowska 89b, 61-614 Poznan, Poland; Department of Cell Biology, Faculty of Biology, Adam Mickiewicz University, Umultowska 89, 61-614 Poznan, Poland; Institute of Molecular Physics, Polish Academy of Sciences, M. Smoluchowskiego 17, 60-179 Poznan, Poland

**Keywords:** Nanoparticles, Erythrocytes, Cytotoxicity, Luminescence, Magnetism, Lanthanides

## Abstract

Multifunctional nanoparticles exhibiting red or green luminescence properties and magnetism were synthesized and thoroughly analyzed. The hydrothermal method was used for the synthesis of Eu^3+^- or Tb^3+^-doped GdF_3_-, NaGdF_4_-, and BaGdF_5_-based nanocrystalline materials. The X-ray diffraction patterns of the samples confirmed the desired compositions of the materials. Transmission electron microscope images revealed the different morphologies of the products, including the nanocrystal sizes, which varied from 12 nm in the case of BaGdF_5_-based nanoparticles to larger structures with dimensions exceeding 300 nm. All of the samples presented luminescence under ultraviolet irradiation, as well as when the samples were in the form of water colloids. The highest luminescence was observed for BaGdF_5_-based materials. The obtained nanoparticles exhibited paramagnetism along with probable evidence of superparamagnetic behavior at low temperatures. The particles’ magnetic characteristics were also preserved for samples in the form of a suspension in distilled water. The cytotoxicity studies against the human erythrocytes indicated that the synthesized nanoparticles are non-toxic because they did not cause the red blood cells shape changes nor did they alter their membrane structure and permeabilization.

**Electronic supplementary material:**

The online version of this article (doi:10.1007/s11051-015-3191-2) contains supplementary material, which is available to authorized users.

## Introduction

In recent decades, nanotechnology and engineering of advanced nanomaterials became intensively researched subjects (Gupta and Gupta 2005; Wang et al. 2005, 2013; Sharma et al. 2006; Lin et al. 2012; Park et al. 2012; Gnach and Bednarkiewicz 2012). Currently, this trend has resulted in research results from numerous applications of nanoparticles (Rapaport et al. 2006; Hinklin et al. 2008; Bedekar et al. 2009; Selvan et al. 2009; Li et al. 2014). The basic properties, such as nanometric dimensions of particles or large surface area, as well as more advanced properties: possibility of functionalization, luminescence, magnetism, and even multifunctional properties, are the factors responsible for this growing interest in the application of nanoparticles. One from, the most important group of studied nanomaterials, is based on the properties of the materials containing lanthanide elements, e.g., the main and the most desired features, such as luminescence or magnetism, results from the properties of lanthanide ions (Ln^3+^) (Grzyb et al. 2012). These unique advantages are particularly interesting for nanomedicine in applications such as bioimaging, photodynamic therapy, or drug delivery (Park et al. 2012; Wang et al. 2013; Tu et al. 2013). By developing Ln^3+^-doped nanocrystals, the properties of existing therapeutic, contrast, or imaging agents may be improved. Additional functionalization of nanoparticles is also possible, thereby changing their cytotoxic properties and providing the possibility of further modification (Grzyb et al. 2013a; Runowski et al. 2014).

Nanoparticles in large amounts are released into the environment. Nanoparticles can reach the human body via different routes, especially the respiratory system or digestive tract, and can enter the blood stream. Moreover, the future application of therapeutic nanoparticles is based on intravenous or oral administration. It has been confirmed that nanoparticles may interact with the blood components and are capable of inducing cell membrane perturbations (Šimundić et al. 2013). Among the blood cells, red blood cells (RBCs) are the most abundant (99 %). Therefore, the studies on the interactions of nanoparticles with RBCs are of considerable importance.

The gadolinium-based nanoparticles are potentially used for cell labeling and cell trafficking in vivo via MRI and/or in vivo optical imaging (Modo et al. 2005; Vuu et al. 2005). However, certain gadolinium complexes are unstable and release the free gadolinium ions, which are paramagnetic. Gd^3+^ ions can also characterize cellular toxicity (Siega et al. 2009). It has been reported that cells can act as “sponges” of free Gd^3+^ ions (Cabella et al. 2006). In the patients with advanced reduction in renal function, treatment with the gadolinium-based cyclic contrast agents (Gd-CA) in MRI investigation induces the creation of toxic-free Gd^3+^ ions and generates nephrogenic systemic fibrosis (Morcos and Thomsen 2008; Perazella 2008). In addition, gadolinium toxicity could be used in the cancer treatment. It has been shown that the internalization of free Gd^3+^ ions, released by unstable vitamin B_12_ bioconjugate, in the human immortalized leukemia K562 cells induces decreases of their viability in vitro (Siega et al. 2009). The human RBCs are the model cells in the study of the effect of different chemical substances on the cell membrane structure and function, including nanoparticles (Rothen-Rutishauser et al. 2006; Šimundić et al. 2013). The ability of RBC, as a model of non-phagocytic cell, to undergo shape deformation and hemolysis after interaction with natural and synthetic compounds is widely used to estimate the cytotoxicity of chemical compounds in general (Mrówczyńska and Hägerstrand 2009; Jasiewicz et al. 2014).

Nanoparticles based on Ln^3+^ properties usually exhibit luminescence. Depending on the final application, the luminescence can be achieved by excitation with ultraviolet (UV), visible, or near infrared (NIR) light. As examples of luminescent ions widely used in recently studied nanophosphors, we used Eu^3+^ and Tb^3+^ ions because of their red or green luminescence. Another co-dopant, Ce^3+^ ions, was used as sensitizers for UV light. Studying the spectroscopic properties of Ln^3+^-doped nanoparticles can also elucidate structure alternations, which are especially visible in nanometric crystals. The spectroscopic properties also allowed us to obtain a complete understanding of the physicochemical characteristics of prepared nanomaterials. Additionally, the addition of Eu^3+^ and Tb^3+^ ions results in multifunctionality of synthesized nanoparticles, giving them both luminescent and magnetic properties via the Gd^3+^ ions in the matrix. Currently, such multifunctionality of nanoparticles is a desired property due to the possibility of using them as contrast agents for both magnetic- and optical-based methods (Liong et al. 2008; Selvan et al. 2009; Shanta Singh et al. 2013).

The magnetic properties of fluorides are governed and can be deduced from the electronic structure of lanthanide ions, where the well-localized 4f electrons have a decisive impact. For example, the addition of Tb^3+^ ions, which possess a high-effective magnetic moment, obscures other phenomena, such as crystal field effects and mixed valency, which are characteristic of Ce-based fluorides (Grzyb et al. 2012; Leycuras et al. 1984). In the case of Gd, one can expect very small crystal field anisotropy, as it has no ionic orbital moment, and a fairly high magnetic moment (the effective magnetic moment of free Gd^3+^ is equal 7.94 μ_B_/ion). When taking into account the possible multifunctionality and biological applications, Gd-based fluorides in colloidal forms were also analyzed.

The present study was aimed to describe the structural, spectroscopic, and magnetic properties of gadolinium fluorides doped by Eu^3+^ and Tb^3+^ and co-doped by Ce^3+^ ions, as well as to characterize their interaction with the cell membrane. The effects of synthesized nanoparticles on the human erythrocyte shape and their sedimentation rate were also investigated.

## Experimental

### Materials

The rare earth oxides Gd_2_O_3_, Eu_2_O_3_, Tb_4_O_7_ (99.99 %) and cerium chloride CeCl_3_·6H_2_O (99.9 %) were purchased from Stanford Materials (United States) and ROTH (Germany), respectively. Ba(NO_3_)_3_ (pure p. a., 99 %), NH_4_F (ACS grade, 98 %), and NaF (ACS grade, 99 %) were purchased from Sigma Aldrich, NaBF_4_ (pure p. a., 97 %) was purchased from Alfa Aesar, and HNO_3_ (ultra-pure) and citric acid monohydrate (pure p.a.) were purchased from POCh S.A. All chemicals were used as starting materials without further purification. To obtain the appropriate nitrates, rare earth oxides were dissolved in the concentrated nitric acid. An excess amount of acid was removed by a three-time repeated evaporation of the solutions. The concentrations of prepared rare earth nitrates and also cerium chloride were set to 1 M. Ultra-pure distilled water was used in all experiments.

### Synthesis of nanoparticles

#### BaGdF_5_:2.5 %Ce^3+^,2.5 %Eu^3+^ and BaGdF_5_:2.5 %Ce^3+^,2.5 %Tb^3+^

The barium gadolinium fluoride samples doped by Ce^3+^ and Eu^3+^ or Tb^3+^ ions were synthesized as follows. Stoichiometric amounts (for 2 mmol of product) of Ba(NO_3_)_2_, Gd(NO_3_)_3_, CeCl_3_, and Eu(NO_3_)_3_ or Tb(NO_3_)_3_ were mixed in 30 mL of distilled water. A stoichiometric amount of NH_4_F was dissolved in 30 mL of distilled water under vigorous stirring and added dropwise into the solution containing barium and lanthanides ions. The obtained colloid was transferred to the Teflon-lined vessel and put under microwave-assisted hydrothermal conditions of 180 °C and 40 bar for 8 h. After cooling down, white precipitate was collected, washed several times with water and ethanol, and then dried at 80 °C for 48 h.

#### GdF_3_:2.5 %Ce^3+^, 2.5 %Eu^3+^ and GdF_3_:2.5 %Ce^3+^, 2.5 %Tb^3+^

The gadolinium fluoride nanoparticles doped by Ce^3+^ and Eu^3+^ or Tb^3+^ ions were obtained by mixing stoichiometric amounts (for 2 mmol of the product) of suitable rare earth salts in 100 mL of distilled water. Precipitation of the product was conducted using two methods. In the first method, NH_4_F was used as the precipitating compound. The second method was based on the decomposition of NaBF_4_ in the hydrothermal conditions. The preparation of RE^3+^ solution was followed by adding dropwise 100 mL of NH_4_F or NaBF_4_ solution (120 % of their stoichiometric amount) under vigorous stirring. The reaction with the first compound resulted in the precipitation of fluoride. The addition of NaBF_4_ was neutral for the color and transparency of the reagents solution. The obtained milky or transparent solutions were transferred into a Teflon-lined autoclave. The reaction was conducted for 2 h at 180 °C and at a pressure of 40 bar. The white precipitates obtained in the each reaction were purified several times via centrifugation with water. The white precipitate was dried in vacuum for 48 h.

#### NaGdF_4_:2.5 %Ce^3+^,2.5 %Eu^3+^ and NaGdF_4_:2.5 %Ce^3+^,2.5 %Tb^3+^

In the typical synthesis of 3 mmol of the samples, appropriate solutions of Gd(NO_3_)_3_, CeCl_3_, and Eu(NO_3_)_3_ or Tb(NO_3_)_3_ were mixed together with citric acid in their stoichiometric ratios. The as-prepared mixture was vigorously stirred for 30 min. Subsequently, NaF (625 % excess of stoichiometric amount) was dissolved in water and mixed with the above solution. The mixture was adjusted to pH = 10 by adding NaOH (2 M) solution. After additional stirring for 15 min, the entire transparent solution was transferred into a Teflon-lined autoclave. The reaction was conducted for over 3 h at 180 °C and at a pressure of 40 bar. The white precipitate obtained was purified several times via centrifugation with water. Finally, the precipitate was dried at 80 °C for 48 h.

All synthesized nanopowders obtained were grounded in agate mortar. Colloidal solutions were prepared by ultrasonification of appropriate amount of nanopowders for 1 h and overnight magnetic stirring of the obtained suspension. Next, after sedimentation of the large and agglomerated particles, the supernatant solution was used for the further studies.

### Methods of cytotoxicity analysis

#### Preparation of erythrocytes

The human RBCs, freshly isolated from buffy coats, were obtained from the blood bank. The RBCs were washed three times (3000 rpm, 10 min, +4 °C) in phosphate-buffered saline (PBS—137 mM NaCl, 2.7 mM KCl, 10 mM NaHPO_4,_ 1.76 mM KH_2_PO_4_, and 10 mM glucose, pH 7.4). After washing, the RBCs were suspended in the buffer at 1.65 × 10^9^ cells/mL, stored at +4 °C, and then used within 5 h.

#### Erythrocyte sedimentation under nanoparticles

The erythrocytes (1.65 × 10^8^ cells/mL) were incubated with nanoparticles at the final concentrations of 0.05, 0.5, 1.0, and 5.0 mg/mL in EP vials for 1 h at 37 °C. The RBCs incubated in PBS only were taken as the control. Each sample was prepared in triplicate, and the experiments were repeated three times using RBCs from different donors. The erythrocytes sedimentation rate (ESR) was recorded using camera. The shapes of both treated and control RBC were estimated using a light microscope. After observation, the RBCs were fixed with 5 % paraformaldehyde plus 0.01 % glutaraldehyde for 1 h at room temperature (RT).

#### Erythrocytes shape under nanoparticles examined using a scanning electron microscope (SEM)

The fixed RBCs were washed three times with PBS (by exchanging supernatant with PBS) and post-fixed with 1 % OsO_4_ for 30 min at RT. Following washing, the RBCs were dehydrated in a series of ethanol solutions (50, 60, 70, 80, 90, 95, and 100 %), critical-point dried, and gold-sputtered. A large number of RBCs were examined using an *EVO 40* (ZEISS, Germany) scanning electron microscope. The SEM micrographs enabled counting of RBCs of varying shape under nanoparticles treatment, and the percentage share of the different forms of RBCs (discocytes, echinocytes, and stomatocytes) in 500 cells was determined, according to the procedure of Bonarska-Kujawa et al. (2012).

#### Ultrathin erythrocyte section observed using a transmission electron microscope (TEM)

The erythrocytes (1.65 × 10^8^ cells/mL) were incubated with nanoparticles at the final concentrations of 5.0 mg/mL in EP vials for 1 h at 37 °C. The RBCs incubated in PBS only were taken as the control. After incubation, the erythrocytes were fixed with 1 % glutaraldehyde in PBS buffer for 1 h at RT. The fixed RBCs were washed three times with PBS (by exchanging supernatant with PBS) and post-fixed with 1 % OsO_4_ in PBS buffer for 1 h at RT. Following washing, the RBCs were dehydrated in a series of ethanol solutions (50, 60, 70, 80, 90, 95, and 100 %). Finally, the RBCs were embedded in Epon 812 with 2 % DMP-30. Ultrathin sections were contrasted with uranyl acetate and lead citrate, and then examined under a JEM 1200 EX II transmission electron microscope.

#### Erythrocytes and nanoparticles detection using a fluorescence microscope

The erythrocytes (1.65 × 10^8^ cells/mL) were incubated with nanoparticles at the final concentrations of 0.05, 0.5, 1.0, and 5.0 mg/mL in EP vials for 1 h at 37 °C. The RBCs incubated in PBS only were taken as the control. Each sample was prepared in triplicate, and the experiments were repeated three times using RBCs from different donors. After incubation, the RBCs were fixed with 5 % paraformaldehyde plus 0.01 % glutaraldehyde for 1 h at RT. Following washing, the RBCs were settled on polylysine-treated (0.1 mg/mL, 10 min) cover glasses and then washed. The cells were mounted on 80 % glycerol. The cover slips were sealed with nail polish. A large number of RBCs in several separate experimental samples were studied for nanoparticle binding using a Zeiss LSM 510 (AXIOVERT ZOOM) fluorescence microscope (100 ×/1.4 aperture immersion oil objective, 10 × ocular) with the appropriate optical filters (for the argon laser wavelength of 488 nm: LP 505 nm or BP 565–615 nm; for the argon laser wavelength of 458 nm: BP 480–520 nm). Images (single-section) were acquired using the Zeiss LSM Image Browser program.

#### Hemolysis assay under nanoparticles

Each RBC suspension (1.65 × 10^8^ cells/mL, ~1.5 % hematocrit) with nanoparticles at different final concentrations (0.05, 0.5, 1.0, and 5 mg/mL) was incubated in EP for 1 h at 37 °C. Each sample was prepared in triplicate, and the experiments were repeated three times with erythrocytes from different donors. After incubation, the RBC suspensions were centrifuged at 3000 rpm for 10 min. The degree of hemolysis (hemoglobin escape from the cell to outer solution) was estimated by measuring the absorbance of the supernatant at 540 nm, as previously reported (Jasiewicz et al. 2014). The absorbance of the control RBC (in PBS only) was used as the blank.

### Characterization

X-ray diffraction (XRD) patterns were collected on a Bruker AXS D8 Advance diffractometer in Bragg–Brentano geometry, with Cu-K_*α*1_ radiation (*λ* = 1.5406 Å) in the 2*θ* range from 6° to 60°. Transmission electron microscopy (TEM) images were collected on the FEI Tecnai G2 20 X-TWIN transmission electron microscope, which used an accelerating voltage of 200 kV. Maud 2.55 software was used to perform Rietveld refinement of cell parameters (Lutterotti and Bortolotti 2003).

The excitation and emission spectra measurements were performed on a Hitachi F-7000 fluorescence spectrophotometer at room temperature. Excitation and emission spectra were corrected for the instrumental response. The QuantaMasterTM 40 spectrophotometer equipped with an Opolette 355LD UVDM tunable laser, which had a repetition rate of 20 Hz as the excitation source and a Hamamatsu R928 photomultiplier as a detector was used to measure luminescence decays.

Magnetic measurements were performed using a quantum design physical property measurement system (PPMS) with a vibrating sample magnetometer (VSM) option at temperatures between 2 and 300 K and in external magnetic fields up to 5 T. The colloid was immured in non-magnetic plastic container and then measured under the same conditions using a quantum design magnetic property measurement system (MPMS).

## Results and discussion

The hydrothermal method has become one of the most promising methods of nanomaterials synthesis (Yoshimura and Byrappa 2007). This convenient approach for synthesis allows for obtaining nanocrystals of different morphologies and is a good alternative for reactions in toxic organic solvents or with the use of annealing and thermolysis. Reactions performed in water at elevated temperature and pressure usually resulted in products of high crystallinity, which is especially important for luminescent nanomaterials, where defects of structure could increase non-radiative transitions and therefore lower the emission efficiency (Karbowiak et al. 2005).

### Structure and morphology

**Fig. 1:**
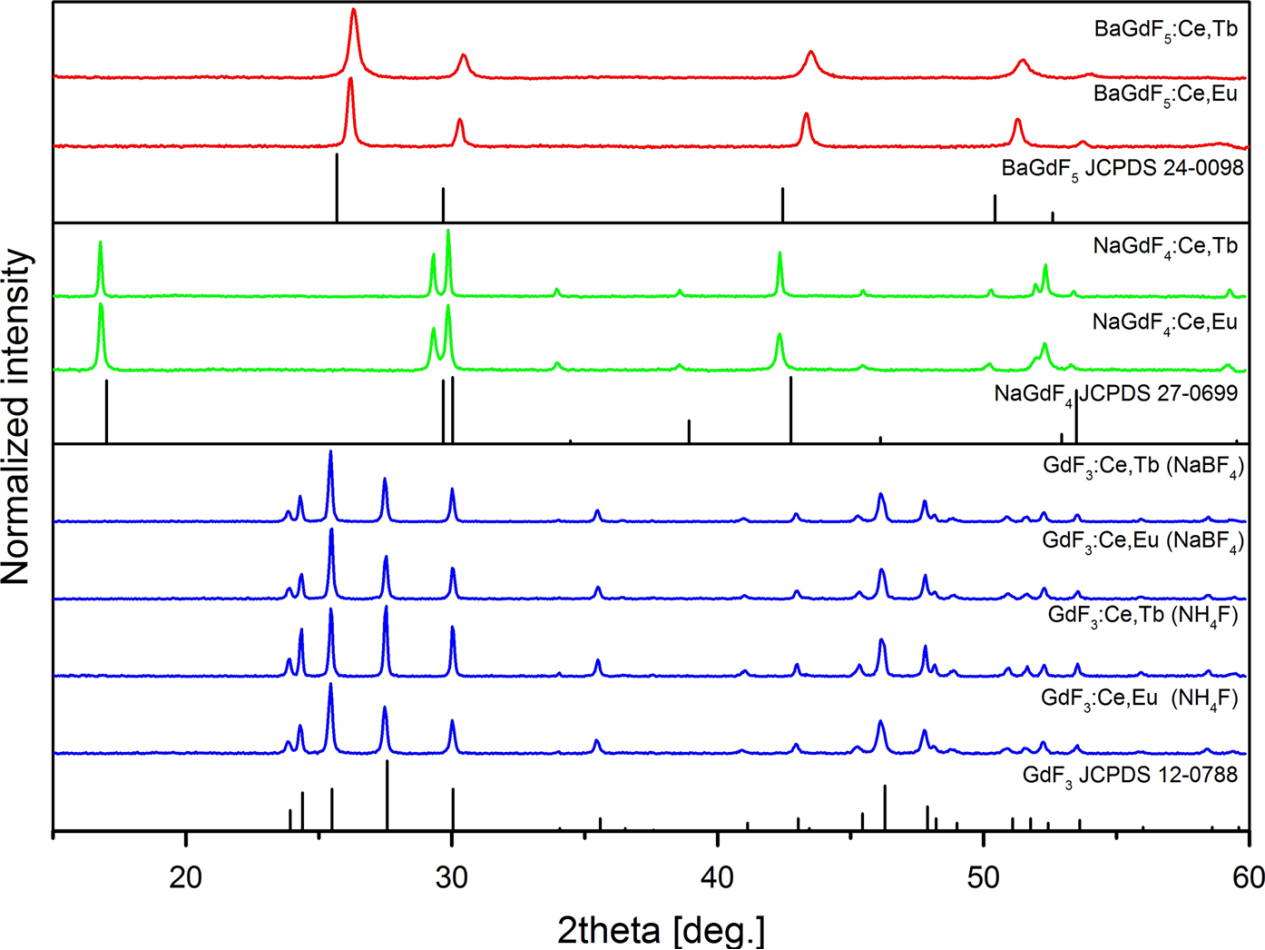
XRD patterns of the BaGdF_5_, NaGdF_4_, and GdF_3_ doped with 2.5 % Ce^3+^ and either 2.5 % Tb^3+^ or 2.5 % Eu^3+^ ions synthesized by hydrothermal method at 180 °C for 2 h

**Fig. 2:**
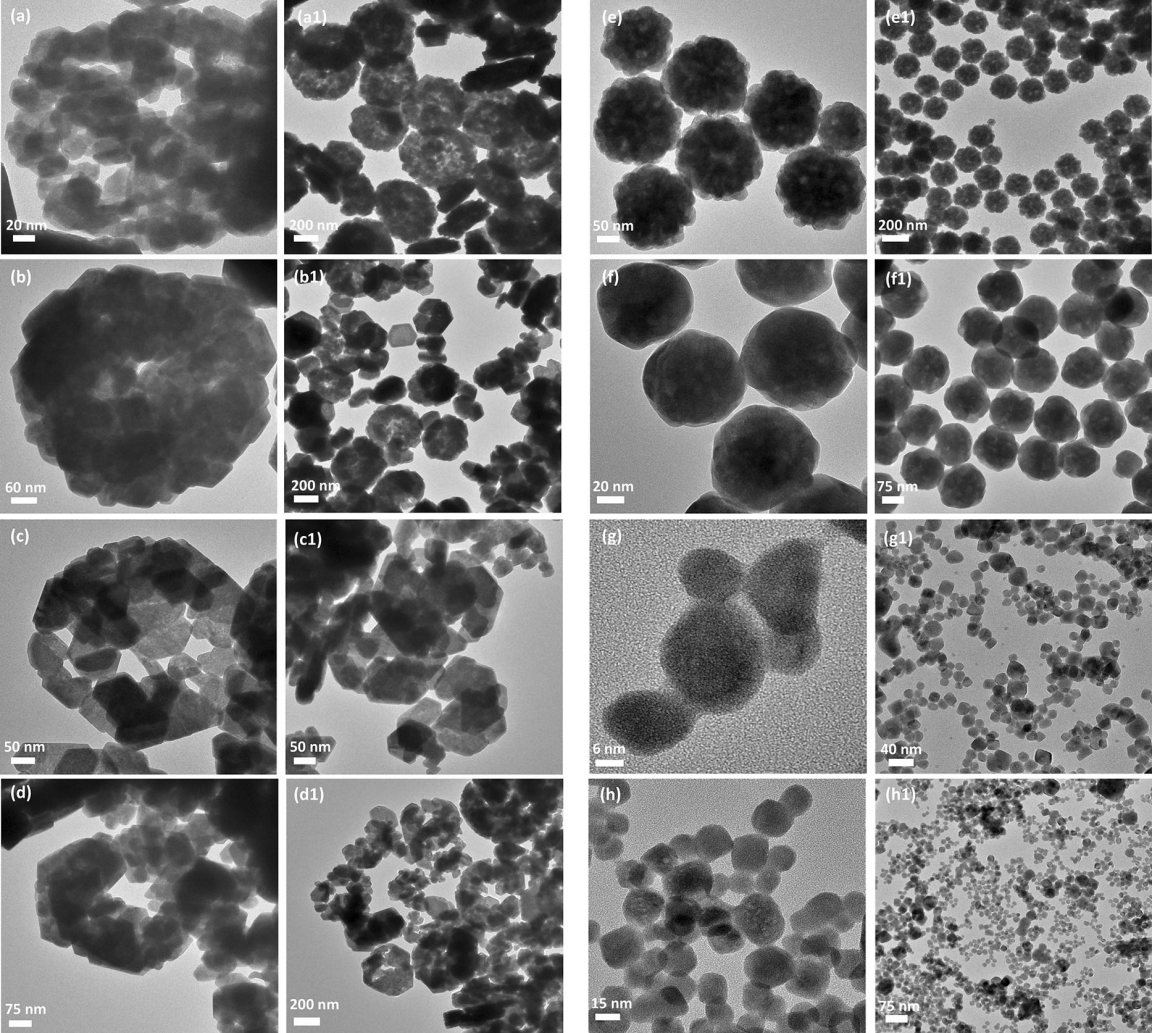
TEM images of the as-synthesized nanomaterials: GdF_3_:2.5 %Ce^3+^,2.5 %Eu^3+^ *(NH*_4_ *F)* (**a**, **a1**), GdF_3_:2.5 %Ce^3+^,2.5 %Tb^3+^ *(NH*_4_ *F)* (**b**, **b1**), GdF_3_:2.5 %Ce^3+^,2.5 %Eu^3+^ *(NaBF*_*4*_ *)* (**c**, **c1**), GdF_3_: 2.5 %Ce^3+^,2.5 %Tb^3+^ *(NaBF*_*4*_ *)* (**d**, **d1**), NaGdF_4_:2.5 %Ce^3+^, 2.5 %Eu^3+^ (**e**, **e1**), NaGdF_4_:2.5 %Ce^3+^,2.5 %Tb^3+^ (**f**, **f1**), BaGdF_5_:2.5 %Ce^3+^, 2.5 %Eu^3+^ (**g**, **g1**), and BaGdF_5_:2.5 %Ce^3+^,2.5 %Tb^3+^ (**h**, **h1**)

The structural analysis of the prepared rare earth fluorides was performed using XRD measurements. Figure 1 presents XRD patterns of the as-prepared BaGdF_5_, NaGdF_4_, and GdF_3_ nanocrystals doped with either Ce^3+^ and Tb^3+^ ions or Ce^3+^ and Eu^3+^ ions. Diffraction patterns for each material exhibit peak broadening, which indicates the nanodimensional size of the crystallites. The most broadened lines are observed for the Ln^3+^-doped BaGdF_5_ samples, which correspond to the smallest average crystallite size among the synthesized materials and are consistent with the TEM images (Fig. 2g, g1, h, and h1). For the Ln^3+^-doped GdF_3_ nanocrystals, all the diffraction peaks clearly demonstrate the presence of orthorhombic GdF_3_ crystal structure, and the *Pnma* space group corresponds to JCPDS No. 12-0788. The most intense reflex for the GdF_3_ samples is (020), which is different in relation to reference data; this difference is an effect of the crystal growth into the preferential orientation (Li et al. 2011). The use of NaBF_4_ has not strongly influenced the structure and morphology of the obtained nanocrystallites. Sodium gadolinium fluoride, NaGdF_4_, can exist in two phases: cubic (*α*-phase) and hexagonal (*β*-phase) (Naccache et al. 2009). The XRD patterns of the Ln^3+^-doped NaGdF_4_ exhibit diffraction peaks that correspond to the presence of the pure hexagonal phase with a space group $P\bar{6}$ (JCPDS No. 27-0699). The diffraction peaks recorded for the BaGdF_5_ nanocrystals can be indexed as cubic phase and space group *Fm*3 *m* (JCPDS No. 24-0098) (Yang et al. 2011).

Note that shifts of the XRD peaks are observed in relation to the reference patterns what resulted from the changed crystal cell volumes. The calculated cell volumes are summarized in Table 1. For the NaGdF_4_ and GdF_3_ used hosts, the calculated cell volume of synthesized compound is larger than this from the reference data. This indicates a nanocrystallinity of obtained materials as the cell volumes of materials in their nanocrystalline form are usually larger than in bulk counterparts. This behavior is known as “size effect” and it is related to defects of the structure and the formation of positive pressure inside the nanocrystal, extending the structure (Ayyub et al. 1995; Tallant et al. 2002). The difference between reference cell volume and this calculated for BaGdF_5_ doped by Ce^3+^ and Tb^3+^ or Eu^3+^ co-dopants is larger than in the remaining compounds. Additionally, the cell volume decreased instead of expected expansion of crystal cell. Addition of dopants into the structure of BaGdF_5_ affected the crystal structure and caused compression of cell volume to similar size as in the case of BaYF_5_ (204.34 Å^3^). The intensities and positions of the peaks for the all nanostructures are in accordance with the literature reference patterns. In addition, sharp and well-resolved peaks indicate the high crystallinity of the samples obtained.

**Table 1:** Cell parameters of synthesized samples compared with references from the JCPDS database

Sample	*a* (Å)	*b* (Å)	*c* (Å)	*V* (Å^3^)
BaGdF_5_^a^	6.023	–	–	218.49
BaGdF_5_:Ce,Tb	5.874	–	–	202.70
BaGdF_5_:Ce,Eu	5.899	–	–	205.27
NaGdF_4_^b^	6.020	–	3.601	113.02
NaGdF_4_:Ce,Tb	6.090	–	3.628	116.55
NaGdF_4_:Ce,Eu	6.088	–	3.633	116.60
GdF_3_^c^	6.571	6.985	4.393	201.63
GdF_3_:Ce,Tb (NaBF_4_)	6.576	6.997	4.404	202.67
GdF_3_:Ce,Eu (NaBF_4_)	6.573	6.993	4.398	202.17
GdF_3_:Ce,Tb (NH_4_F)	6.572	6.995	4.400	202.26
GdF_3_:Ce,Eu (NH_4_F)	6.575	6.999	4.406	202.77

^a^JCPDS 24-0098

^b^JCPDS 27-0699

^c^JCPDS 12-0788

The size and morphology of the nanomaterials obtained were characterized based on TEM images (Fig. 2a–h, taken with different magnifications). The products are well crystallized and exhibit interesting and different features for each fluoride morphology. The GdF_3_:Ce^3+^,Eu^3+^ and GdF_3_:Ce^3+^,Tb^3+^ samples synthesized with the presence of NH_4_F and NaBF_4_ as a fluorine source have similar morphology and tend to form aggregates with the dimensions of approximately 300 nm. These structures are constructed with many self-assembled nanocrystallites of size not exceeding 20 nm. The reports of the synthesis of similar structures can be found in the literature (Wang et al. 2006; Zhong et al. 2009; Safronikhin et al. 2011; Grzyb et al. 2013b). The morphology of GdF_3_:Ln^3+^ nanocrystals can be described as distorted or not fully formed rings. The formation mechanism of ring-like structures was explained previously and involves the crystallization of hexagonal particles at the initial step (Zhong et al. 2009). In the next step, a phase transition to the orthorhombic arc-like crystals occurs, followed by their aggregation to ring-like structures.

Figure 2e, e1, f, and f1 shows TEM images of the as-prepared Ln^3+^-doped NaGdF_4_ samples. From the high-magnification TEM image, monodisperse and uniform nanospheres (average size is of approximately 150 nm) are found to contain many small nanocrystals of size of approximately 10 nm, which are more densely packed in the formed aggregates compared to Ln^3+^-doped GdF_3_. This result indicates the possible self-assembly process during the synthesis (He et al. 2011).

The morphology of the BaGdF_5_-based nanocrystals differs from the remaining samples (Fig. 2g, g1, h, and h1). The nanocrystals exhibit a quite regular shape and an average grain size of approximately 15 nm. The presented materials are composed of monocrystals, in contrast to the polycrystalline particles in the case of GdF_3_:Ln^3+^ or NaGdF_4_:Ln^3+^.

### Spectroscopic properties

**Fig. 3:**
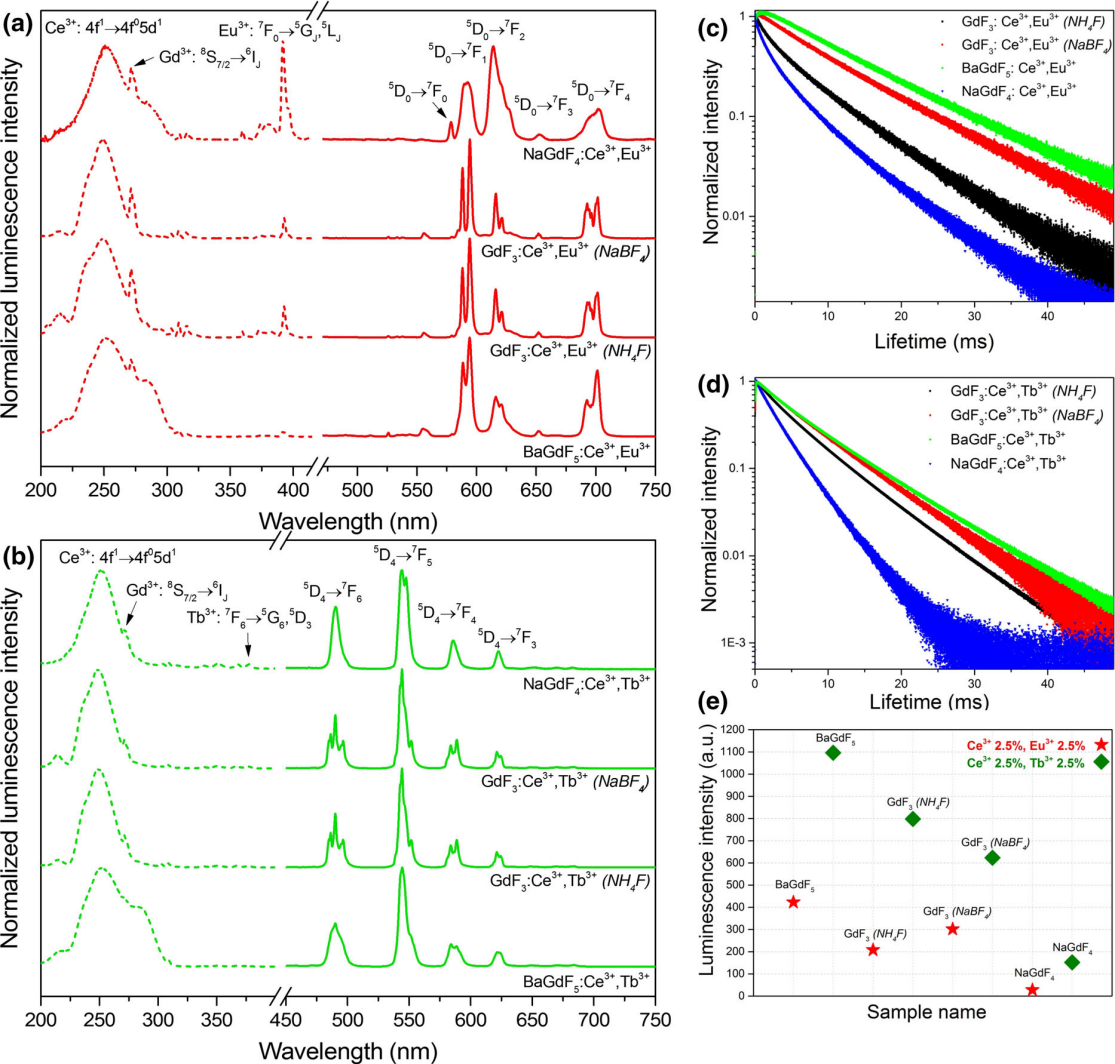
Excitation (*dashed lines*) and emission (*solid lines*) spectra (**a**, **b**), luminescent lifetimes (**c**, **d**) and emission intensities (**e**) of the fluorides obtained, doped with 2.5 %Ce^3+^,2.5 %Eu^3+^ and 2.5 %Ce^3+^,2.5 %Tb^3+^. For the Eu^3+^-doped samples, the excitation wavelength used was *λ*_ex_ = 253 nm, and the observed emission wavelength was *λ*_em_ = 592 nm; for the Tb^3+^-doped samples, the excitation wavelength was *λ*_ex_ = 253 nm, and the emission wavelength was *λ*_em_ = 543 nm

The nanomaterials obtained exhibited intensive red luminescence because of the energy transfer (ET) phenomenon that occurred between the dopant ions. In the synthesized compounds, Ce^3+^ ions acted as luminescence sensitizers for UV radiation (energy donors) and Gd^3+^ ions acted as energy mediators, which transfer energy to the appropriate Ln^3+^ activator ion (Eu^3+^ or Tb^3+^) (Grzyb et al. 2014). The compounds doped with Eu^3+^ ions exhibited red luminescence, and the compounds doped with Tb^3+^ ions exhibited green luminescence. The total emission intensity was dependent on the activator ion used, as well as on the host composition (see Fig. 3e). Generally, the compounds exhibiting green luminescence (doped with Tb^3+^ ions) had higher total emission intensity in comparison to that of the red nanophosphors (doped with Eu^3+^ ions). This phenomenon was related to the more efficient luminescence quenching of Eu^3+^ ions and the more effective energy transfer to the Tb^3+^ (the energy gap between Gd^3+^ and Tb^3+^ ions is smaller than in the case of Gd^3+^ and Eu^3+^ ions). In addition, the compounds based on BaGdF_5_ hosts exhibited the most intense emission, and the phosphors based on NaGdF_4_ revealed the lowest luminescence intensity. This result occurred because of the impacts of the selected crystal structure, grain size, and crystallinity.

The excitation and emission spectra of the nanomaterials synthesized are presented in Fig. 3a, b. The spectra were recorded at room temperature, in ambient conditions. The measured spectra were normalized to the intensity of the most intense band. Figure 3a shows the excitation (dashed lines) and emission spectra (solid lines) of Eu^3+^-doped fluorides. The excitation spectra were measured at *λ*_em_ = 592 nm (the most intense band related to the $^{ 5} D_{0} \to ^{ 7} F_{ 1}$ transition in Eu^3+^ ion). The intense and very broad band in the range of 220–300 nm was assigned to the $4f^{ 1} \to 4f^{0} 5d^{ 1}$ transition in Ce^3+^ ion, confirming the above-mentioned ET phenomenon (ET from Ce^3+^ to Gd^3+^ and Eu^3+^). At 272 nm, another intense band was observed, which is related to the $^{ 8} S_{ 7/ 2} \to ^{ 6} I_{J}$ transition of Gd^3+^ ions, thereby confirming the ET from Gd^3+^ to Eu^3+^ ions. The narrow bands in the range of 300–400 nm correspond to the $4f^{ 8} - 4f^{ 8}$ transitions in Eu^3+^ ions.

The emission spectra recorded at *λ*_ex_ = 253 nm at the maximum of the most intense excitation band are related to the $4f^{ 1} \to 4f^{0} 5d^{ 1}$ transition of the Ce^3+^ ion. In these spectra, six narrow bands corresponding to the $^{ 5} D_{ 1} \to ^{ 7} F_{J} \left( {J = 0{-} 4} \right)$ transitions of Eu^3+^ ions were observed. These bands are typical for Eu^3+^ ions and result in the red–orange luminescence of the obtained materials. In addition, for NaGdF_4_:Ce^3+^, Eu^3+^, the intensity ratios of the recorded bands are similar for the obtained compounds, suggesting that Eu^3+^ ions have similar local environment in both the GdF_3_ and BaGdF_5_ fluoride hosts. The hyperfine $^{ 5} D_{0} \to ^{ 7} F_{ 2}$ electric dipole transition (at approximately 615 nm) is less intense than the $^{ 5} D_{0} \to ^{ 7} F_{ 1}$ (≈ 592 nm) magnetic dipole transition, which confirms the centrosymmetric local coordination of the Eu^3+^ ion environment in the synthesized nanocrystals (Tanner 2013). The Eu^3+^ ions occupy sites with *C*_*s*_ symmetry in GdF_3_ and *C*_4*v*_ in BaGdF_5_. (Wells et al. 2002; Guo et al. 2012) However, in the case of the NaGdF_4_ compound, the mentioned $^{ 5} D_{0} \to ^{ 7} F_{ 2}$ transition is more intense than the $^{ 5} D_{0} \to ^{ 7} F_{ 1}$ transition, which indicates that Eu^3+^ ions occupy a site without inversion symmetry. Indeed, in the NaGdF_4_ host, Eu^3+^ ions occupy sites with *C*_3*h*_ symmetry (Karbowiak et al. 2005).

The excitation spectra of Tb^3+^-doped compounds were measured at *λ*_em_ = 543 nm (dashed lines in Fig. 3b). The excitation spectra of Tb^3+^-doped compounds are similar to the Eu^3+^ spectra because of the presence of a wide and intense band related to the absorption of light by Ce^3+^ ions, the narrow bands related to the presence of Gd^3+^ ions, and a series of less intense $4f - 4f$ transition bands of Tb^3+^ ions. However, the $^{ 8} S_{ 7/ 2} \to ^{ 6} I_{J} \left( {{\text{Gd}}^{ 3+ } } \right)$ and $4f - 4f\left( {{\text{Eu}}^{ 3+ } } \right)$ transitions reveal lower intensity in comparison to the broad $4f^{ 1} \to 4f^{0} 5d^{ 1}$ transition in the Ce^3+^ ion. This difference is due to the more effective ET from Ce^3+^ to Tb^3+^ ions.

The emission spectra were recorded at the same *λ*_ex_ = 253 nm (solid lines in Fig. 3b). In all the spectra, the four narrow bands typical of Tb^3+^ ions can be observed. These bands are related to the ^5^*D*_4_ → ^7^*F*_*J*_ (*J* = 6–3) transitions of Tb^3+^ ions. The intensity ratios of the mentioned bands are similar in all of the obtained products because all of them correspond to magnetic dipole transitions (insensitive to site symmetry). The presence of these bands results in the green emission of the obtained nanomaterials.

The spectroscopic analysis was completed by performing measurements of the decay curves of the nanophosphors obtained, presented in Fig. 3c, d. All the values of the luminescent lifetimes range from 1.2 to 14.4 ms are presented in Table 2. The decay curves were obtained by monitoring the $^{ 5} D_{0} \to ^{ 7} F_{1} \left( {\lambda_{\text{em}} = 5 9 2 {\text{nm}}} \right)$ and $^{ 5} D_{ 4} \to ^{ 7} F_{5} \left( {\lambda_{\text{em}} = 5 4 3 {\text{nm}}} \right)$ transitions for the Eu^3+^- and Tb^3+^-doped fluorides, respectively. The luminescence decays varied from the single-exponential to bi-exponential, indicating the presence of a non-radiative process. Therefore, the effective lifetimes were calculated using the following equation (Lakowicz 2006):

**Table 2:** Calculated luminescent lifetimes of the synthesized Eu^3+^ and Tb^3+^-doped fluorides

Sample	*τ*_*1*_ [ms]
GdF_3_:2.5 %Ce^3+^,2.5 %Eu^3+^ *(NH*_*4*_ *F)*	5.6 ± 0.1
GdF_3_:2.5 %Ce^3+^,2.5 %Eu^3+^ *(NaBF*_*4*_ *)*	11.0 ± 0.1
NaGdF_4_:2.5 %Ce^3+^,2.5 %Eu^3+^	3.4 ± 0.1
BaGdF_5_:2.5 %Ce^3+^,2.5 %Eu^3+^	14.4 ± 0.1
GdF_3_:2.5 %Ce^3+^,2.5 %Tb^3+^ *(NH*_*4*_ *F)*	5.6 ± 0.1
GdF_3_: 2.5 %Ce^3+^,2.5 %Tb^3+^ *(NaBF*_*4*_ *)*	6.8 ± 0.1
NaGdF_4_: 2.5 %Ce^3+^,2.5 %Tb^3+^	3.2 ± 0.1
BaGdF_5_: 2.5 %Ce^3+^,2.5 %Tb^3+^	7.1 ± 0.1

$$\tau = \frac{{\int\limits_{0}^{\infty } {tI(t){\text{d}}t} }}{{\int\limits_{0}^{\infty } {I(t){\text{d}}t} }}$$

The calculated lifetime values for Eu^3+^-doped samples are comparable with other reported values (Karbowiak et al. 2005; Grzyb et al. 2014). The quenching processes are strongly dependent not only on the host material used but also on the substrates used for the synthesis. When NaBF_4_ is used as the fluorine source, the obtained material exhibits a longer luminescence lifetime. Most probably, Na^+^ ions provide some alternations in the crystal structure, resulting in the lower quenching of Eu^3+^ excited states. Differences between host materials are reasonable, considering the different morphologies of the products and the local environments of the sites occupied by Eu^3+^ ions. Similar results were obtained in the case of Tb^3+^-doped samples. The use of NaBF_4_ as a reagent decreased the amount of quenching on the Tb^3+^ luminescence. Additionally, BaGdF_5_ appears to be a better host material for the luminescent Ln^3+^ ions. The luminescence lifetime is important when bioimaging applications are considered. Longer emission lifetimes give the possibility of the full separation of the signal from the emission of background.

### Magnetic properties

From the point of view of magnetic properties and possible applications, it was important to show not only the solute behavior but also the characteristics of the entire colloid. Hence, the diamagnetic contribution of water was also taken into account. While changing the temperature and pressure, water undergoes many phase transitions and changes its volume (Stanley et al. 2000). Such changes could influence the magnetic behavior by differing the interparticle distances, especially in the frozen state. Nevertheless, the static picture of a colloid at room temperature is similar to the picture of particles embedded in ice matrix at low temperature. Due to the negligible interparticle magnetic interactions, it is highly probable that the particles are agglomerated in none of mentioned states and that the magnetic properties determined at different temperatures can be directly compared.

**Fig. 4:**
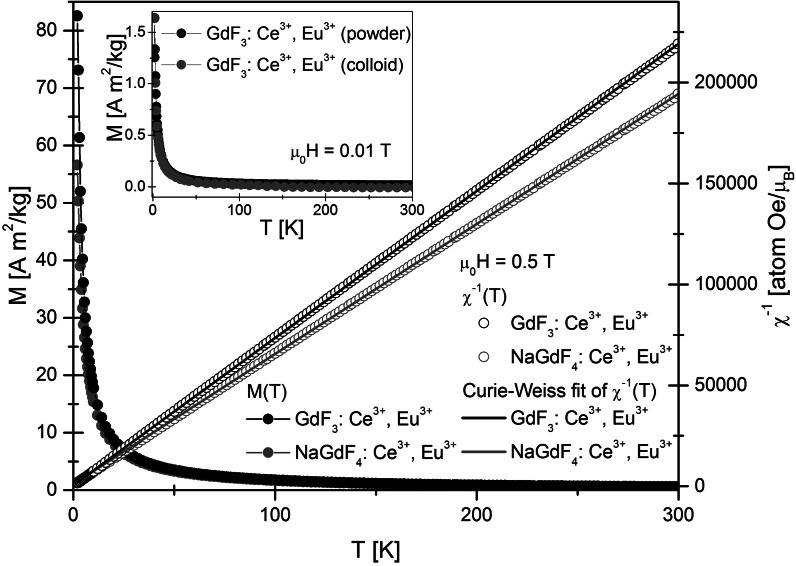
Temperature dependence of the magnetization *M* (*left scale*) and of the reciprocal DC magnetic susceptibility 1/*χ* (*right scale*) in GdF_3_:Ce^3+^,Eu^3+^, and NaGdF_4_:Ce^3+^,Eu^3+^. A comparison of GdF_3_:Ce^3+^,Eu^3+^ in powder and colloidal form is presented in the* inset*

Magnetic measurements were performed for four different powder samples (GdF_3_:Ce^3+^,Eu^3+^, GdF_3_:Ce^3+^,Tb^3+^, NaGdF_4_:Ce^3+^,Eu^3+^, and NaGdF_4_:Ce^3+^,Tb^3+^) and colloid of GdF_3_:Ce^3+^,Eu^3+^ suspended in distilled water. Due to the higher mass susceptibility of GdF_3_:Ce^3+^, one of these powders (with Eu^3+^ substitution) was chosen as the colloid solute. Temperature dependences of magnetization *M(T)* in the zero-field cooled (ZFC) and field cooled (FC) modes were measured in the temperature range of 2–300 K. The ZFC and FC curves were found to overlap each other. The curves measured for GdF_3_ with different substitutions are superimposed, as well as those for both NaGdF_4_-based samples. This result indicates that the influence of the substitution of Eu and Tb on the magnetic properties of the samples is roughly the same. As a consequence, only the results for Ce^3+^- and Eu^3+^-substituted samples are presented in Fig. 4. The results for Tb^3+^ substituted powders are shown in the Supplement (Figs. S1 and S2). Both *M(T)* curves coincide rather well over almost the entire temperature range, and the curves are typical for paramagnets. The magnetic properties of the Gd^3+^, which arise from seven unpaired inner 4*f* electrons, are responsible for such behavior. Gd^3+^ bonded with fluorine ions forms compounds, where the separation between the Gd^3+^ ions does not allow ferromagnetic interactions. In the inset of Fig. 4, the comparison of GdF_3_:Ce^3+^,Eu^3+^ as powder and water dispersed colloid is shown. The magnetization of the colloid was not divided per mass of the magnetic substance, but just rescaled to match the maximum value of the powder magnetization at 2 K. Hence, the mentioned *M(T)* curves should not be compared in a quantitative way. Qualitatively, the paramagnetic properties of GdF_3_:Ce^3+^,Eu^3+^ powder are preserved with increasing interparticle distance in the GdF_3_:Ce^3+^,Eu^3+^ colloid. This result suggests a lack of magnetic interparticle interactions. To expand the analysis of the magnetic data, the temperature dependences of the reciprocal DC magnetic susceptibility were plotted (Fig. 4—right axis). The DC magnetic susceptibility is defined as the quotient of *M* and *H*. Both curves for GdF_3_:Ce^3+^,Eu^3+^ and NaGdF_4_:Ce^3+^,Eu^3+^ follow the Curie–Weiss law, with paramagnetic Curie temperatures *Θ*_*p*_ equal to −1 and −2 K, respectively. The effective magnetic moments *μ*_*eff*_ per magnetic ion are comparable to that of free Gd^3+^ (7.94 μ_B_): 7.83 μ_B_ for GdF_3_:Ce^3+^,Eu^3+^ and 8.13 μ_B_ for NaGdF_4_:Ce^3+^,Eu^3+^. The magnetic mass susceptibilities *χ*_*g*_ at 300 K are equal to 1.19 × 10^−4^ emu/(gOe) and 1.12 × 10^−4^ emu/(gOe) for GdF_3_:Ce^3+^,Eu^3+^ and NaGdF_4_:Ce^3+^,Eu^3+^, respectively. These values are slightly higher than magnetic mass susceptibilities determined for GdF_3_, GdF_3_:Eu^3+^, and NaGdF_4_:Yb^3+^,Er^3+^ (Wong et al. 2009; Wang et al. 2010, 2012). The magnetic behavior of all the GdF_3_ and NaGdF_4_ nanoparticles described above is governed by the independent dynamics of the magnetic moment of each rare earth ion.

**Fig. 5:**
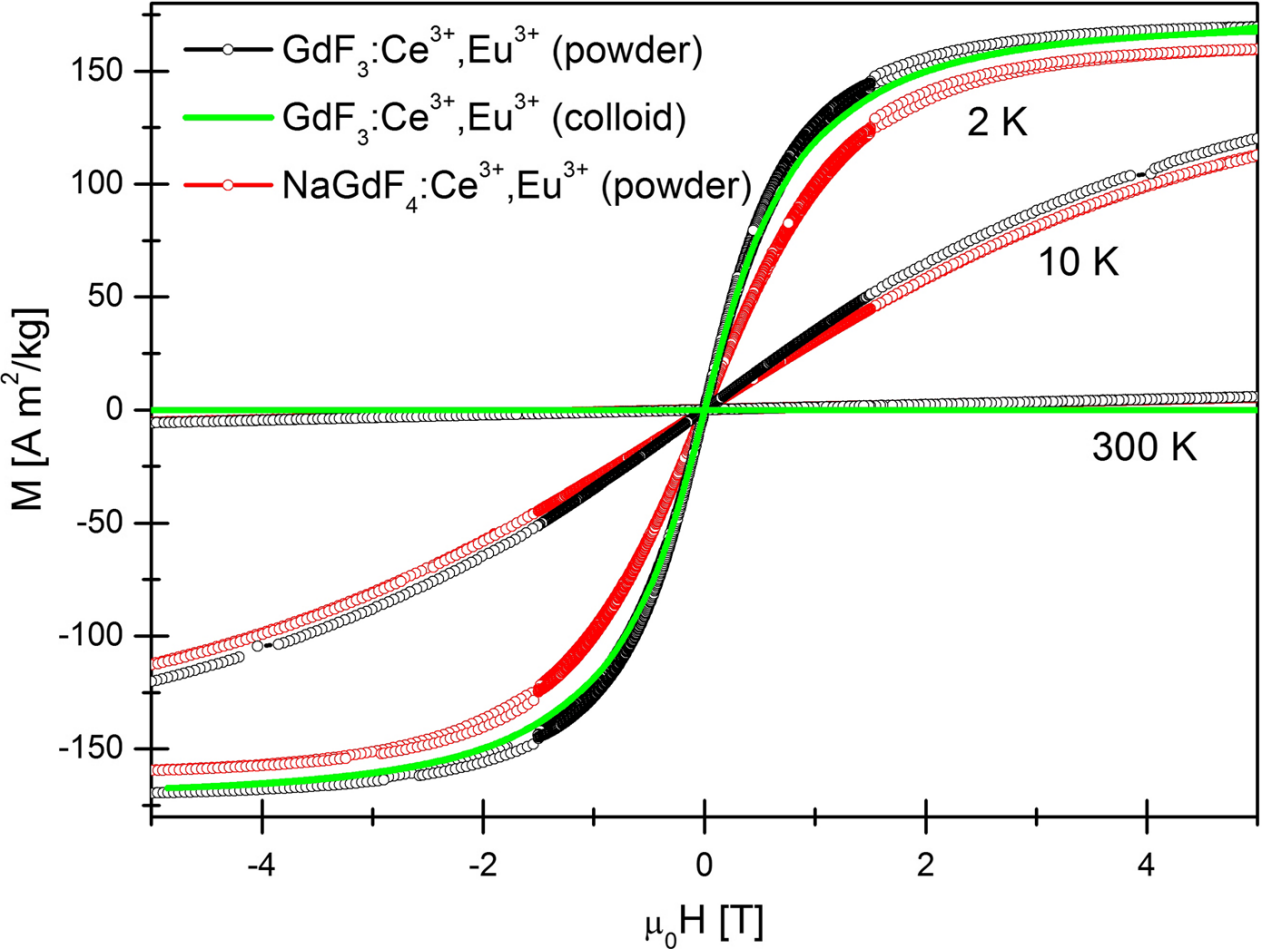
Magnetic field dependence of the magnetization *M* in GdF_3_:Ce^3+^,Eu^3+^ powder, GdF_3_:Ce^3+^,Eu^3+^ colloid, and NaGdF_4_:Ce^3+^,Eu^3+^ powder measured at 2, 10, and 300 K

The magnetic field dependence of the magnetization for powder samples GdF_3_:Ce^3+^,Eu^3+^; GdF_3_:Ce^3+^,Tb^3+^; NaGdF_4_:Ce^3+^,Eu^3+^; and NaGdF_4_:Ce^3+^,Tb^3+^ was measured at 2, 10, and 300 K. Magnetic isotherms for the GdF_3_:Ce^3+^,Eu^3+^ colloid were obtained at 2 and 300 K. The *M(H)* curves for different substitutions (Eu^3+^, Tb^3+^) are very similar, so GdF_3_:Ce^3+^,Tb^3+^ and NaGdF_4_:Ce^3+^,Tb^3+^ are omitted below for clarity (see Fig. S2 in the Supplement). In Fig. 5, the results for GdF_3_:Ce^3+^,Eu^3+^ and NaGdF_4_:Ce^3+^,Eu^3+^ are presented. In addition, the curves measured at 2 and 300 K for GdF_3_:Ce^3+^,Eu^3+^ colloid are shown. There is no saturation of magnetization up to *μ*_0_*H* = 5 T, and the curves for both samples do not exhibit any hysteresis. At 300 K, all samples can be described as typical paramagnets. The low-temperature *M(H)* dependence could indicate superparamagnetism, but ZFC and FC *M(T)* are not observed to deviate from each other, and there is no cusp down to 2 K. This result could indicate an ultralow blocking temperature. The saturation of magnetization reaches 170 Am^2^/kg. There is also strong diamagnetic influence on M(H) of colloid visible at 300 K, which is connected with the water contribution. As in the case of the *M(T)* results, the magnetization of the colloid was rescaled to match the maximum value of the powder magnetization. Again, the results are qualitatively very similar to those of GdF_3_:Ce^3+^,Eu^3+^ powder and do not indicate the presence of any interparticle magnetic interactions. The magnetic behavior does not depend on the interparticle distance. Magnetization at 2 T and 300 K was determined to be equal to 2.32 and 2.17 emu/g for GdF_3_:Ce^3+^,Eu^3+^ and NaGdF_4_:Ce^3+^,Eu^3+^, respectively. The measured values are comparable to the value of 2 emu/g reported previously for GdF_3_ and GdF_3_:Eu^3+^, which potentially qualifies them for use as bioseparation nanoparticles (Wong et al. 2009; Wang et al. 2012).

### Cytotoxicity study of nanoparticles on human erythrocytes

**Fig. 6:**
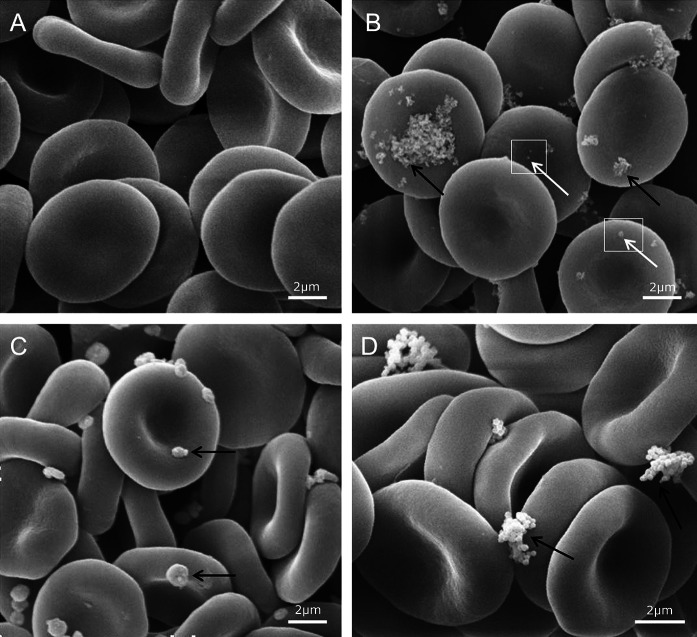
The effect of **a** PBS (control), **b** BaGdF_5_: 2.5 %Ce^3+^, 2.5 %Tb^3+^ **c** GdF_3_: 2.5 %Ce^3+^, 2.5 %Tb^3+^ (NaBF_4_), and **d** NaGdF_4_: 2.5 %Ce^3+^, 2.5 %Tb^3+^ nanoparticles (5 mg/mL, 1 h, 37 °C) on human erythrocytes, as observed using a scanning electron microscope. The* arrows* indicate single (*white arrows*) and aggregated (*black arrows*) nanoparticles attached to the RBC membrane

**Fig. 7:**
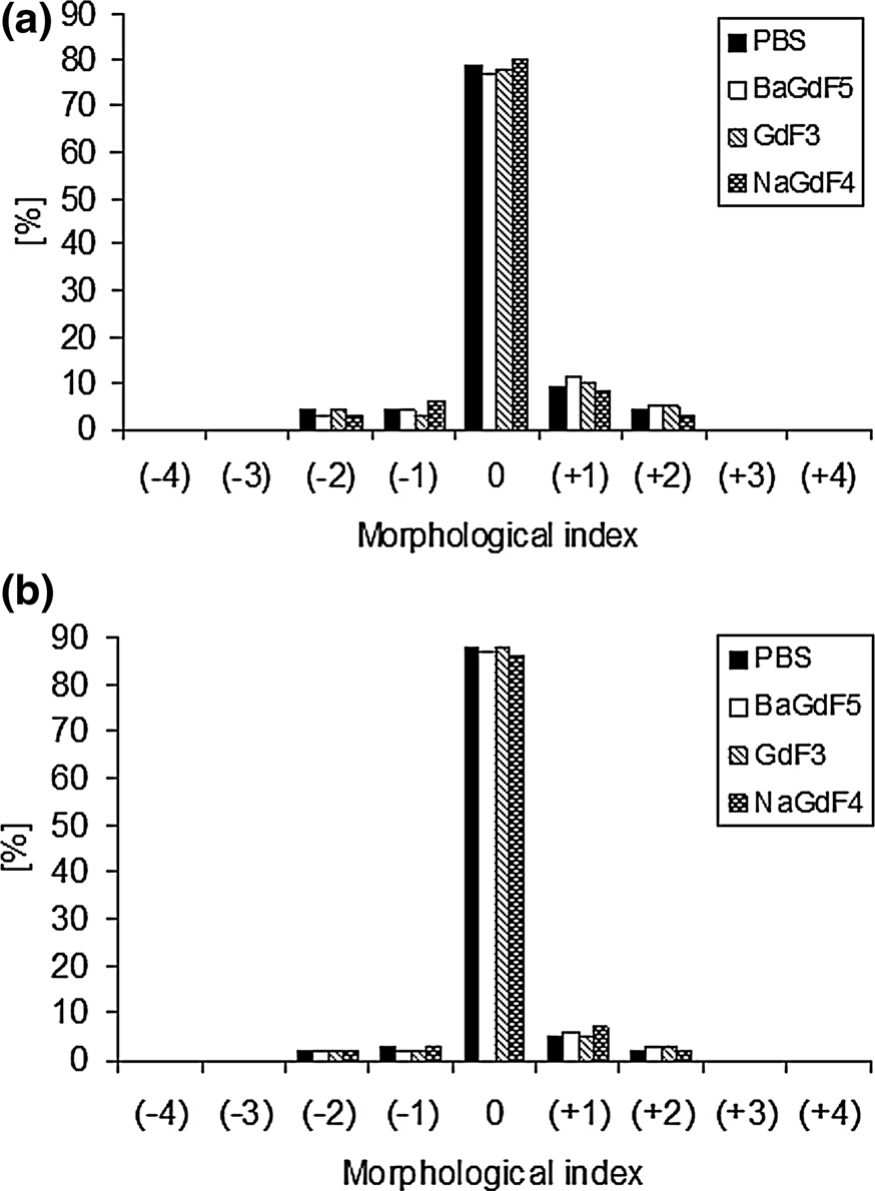
Percent share of different shapes of human erythrocytes in the presence of nanoparticles at the concentration of 5 mg/mL after (**a**) 1 h and (**b**) 12 h incubation at 37 °C. On the abscissa, the morphological indices for the respective shape of RBC are spherostomatocytes (−4), stomatocytes II (−3), stomatocytes I (−2), discostomatocytes (−1), discocytes (0), discoechinocytes (+1), echinocytes (+2), spheroechinocytes (+3), and spherocytes (+4)

The method of cytotoxicity assessment was based on the estimation of the RBC shape changes and measurement of the efflux of hemoglobin from RBCs exposed to nanoparticles, namely, hemolysis. Figure 6 shows the effect of nanoparticles on the RBC shape, as observed using a scanning electron microscope. None of the nanoparticle types used in the study affected the discoid shape of erythrocytes in the concentrations used. Both the control RBCs (Fig. 6a) and the RBCs treated with different nanoparticles (Fig. 6b–d) were discocytes after 1 h incubation (Fig. 7a). Moreover, all nanoparticle types exhibited no effect on the RBC shape after 12 h incubation (Fig. 7b). As shown in Figs. 6b–d, single and aggregated nanoparticles were found to be attached to the RBC membrane. Single nanoparticles were confirmed to have a tendency to aggregate in the aqueous suspension before binding to the cellular membrane (Takenaka et al. 2001; Šimundić et al. 2013). Interestingly, the smaller nanoparticles exhibited higher hemolytic potency compared with their large aggregates (Kim and Shin 2014). The present studies indicate that the analyzed nanoparticles are not toxic, either as single nanocrystals or as nanocrystals aggregates. The RBCs exposed to all types of the analyzed nanoparticles in the concentration range from 0.05 mg/ml to 5 mg/ml did not undergo hemolysis. The percent of hemolysis in the presence of nanoparticles was calculated to be in the range of 0–2 %, as estimated for the control RBC.

**Fig. 8:**
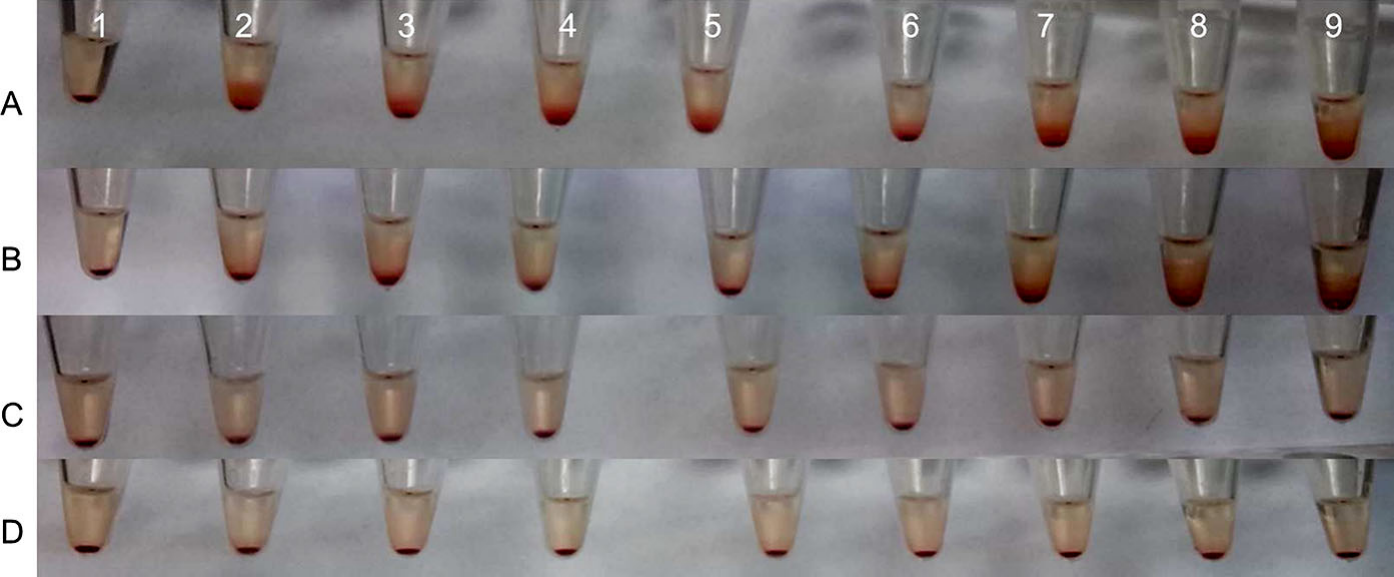
The sedimentation rate of RBSs in the presence of nanoparticles after 1 h at 37 °C. (**a**) 5 mg/mL, (**b**) 1 mg/mL, (**c**) 0.5 mg/mL, and (**d**) 0.05 mg/mL. (*1*) PBS (control), (*2*) NaGdF_4_:2.5 %Ce^3+^,2.5 %Eu^3+^, (*3*) NaGdF_4_:2.5 %Ce^3+^,2.5 %Tb^3+^, (*4*) GdF_3_:2.5 %Ce^3+^,2.5 %Eu^3+^, (*5*) GdF_3_:2.5 %Ce^3+^,2.5 %Tb^3+^, (*6*) GdF_3_:2.5 %Ce^3+^,2.5 %Eu^3+^ (NaBF_4_), (*7*) GdF_3_:2.5 %Ce^3+^,2.5 %Tb^3+^ (NaBF_4_), (*8*) BaGdF_5_:2.5 %Ce^3+^,2.5 %Eu^3+^, and (*9*) BaGdF_5_:2.5 %Ce^3+^, 2.5 %Tb^3+^

The RBCs settled at the same sedimentation rate as the control erythrocytes are at 0.5 and 0.05 mg/mL nanoparticle concentrations (Fig. 8c–d). At the higher concentrations, namely, 1 mg/mL and 5 mg/mL, slight disturbances of the RBC sedimentation were observed (Fig. 8a, b). According to present data, the sedimentation disturbance could be induced by two factors: the colloidal nature of the nanoparticle solutions or by the nanoparticles, especially their aggregates, attached to the RBC membrane. Although the first factor seems to be more probable, the synergic action of both could not be excluded.

**Fig. 9:**
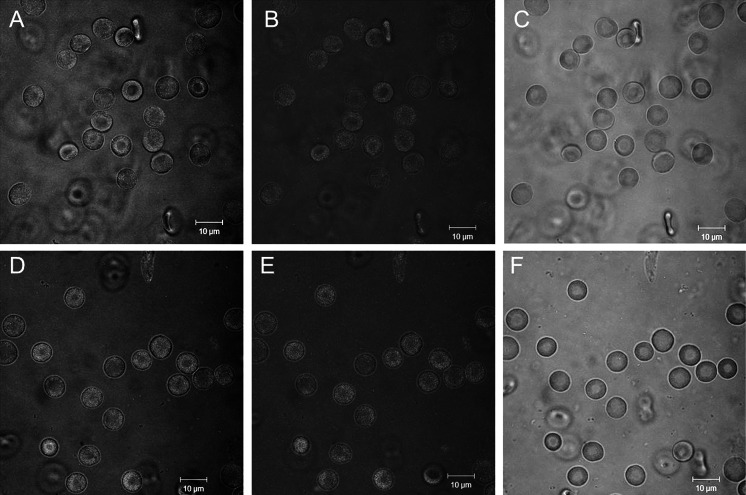
**a**, **b**, **c** The effect of PBS and **d**, **e**, **f** GdF_3_: 2.5 %Ce^3+^,2.5 %Tb^3+^ (NaBF_4_) at 5 mg/mL (1 h, 37 °C) on human erythrocytes, as observed using a fluorescence microscope. (**a** and **d**) argon laser wavelength of 488 and LP505 nm, (**b** and** e**) argon laser wavelength of 488 nm and BP 565–615 nm, (**c** and **f**) argon laser wavelength of 458 nm and BP 480–520 nm. No noticeable changes were observed between the autofluorescence of the control erythrocytes (**a**, **b**, **c**) and the erythrocytes exposed to nanoparticles (**d**, **e**, **f**). *Scale bars* indicate 10 µm

We applied fluorescence microscopic techniques to visualize the nanoparticles attached to the RBC membrane. Unfortunately, the fluorescence signals of the RBCs treated with nanoparticles (Fig. 9d–f) and the RBCs incubated without (Fig. 9a–c) were found to be similar. Therefore, the precise detection of RBC membrane-bound nanoparticles using fluorescence microscopy techniques was not possible.

**Fig. 10:**
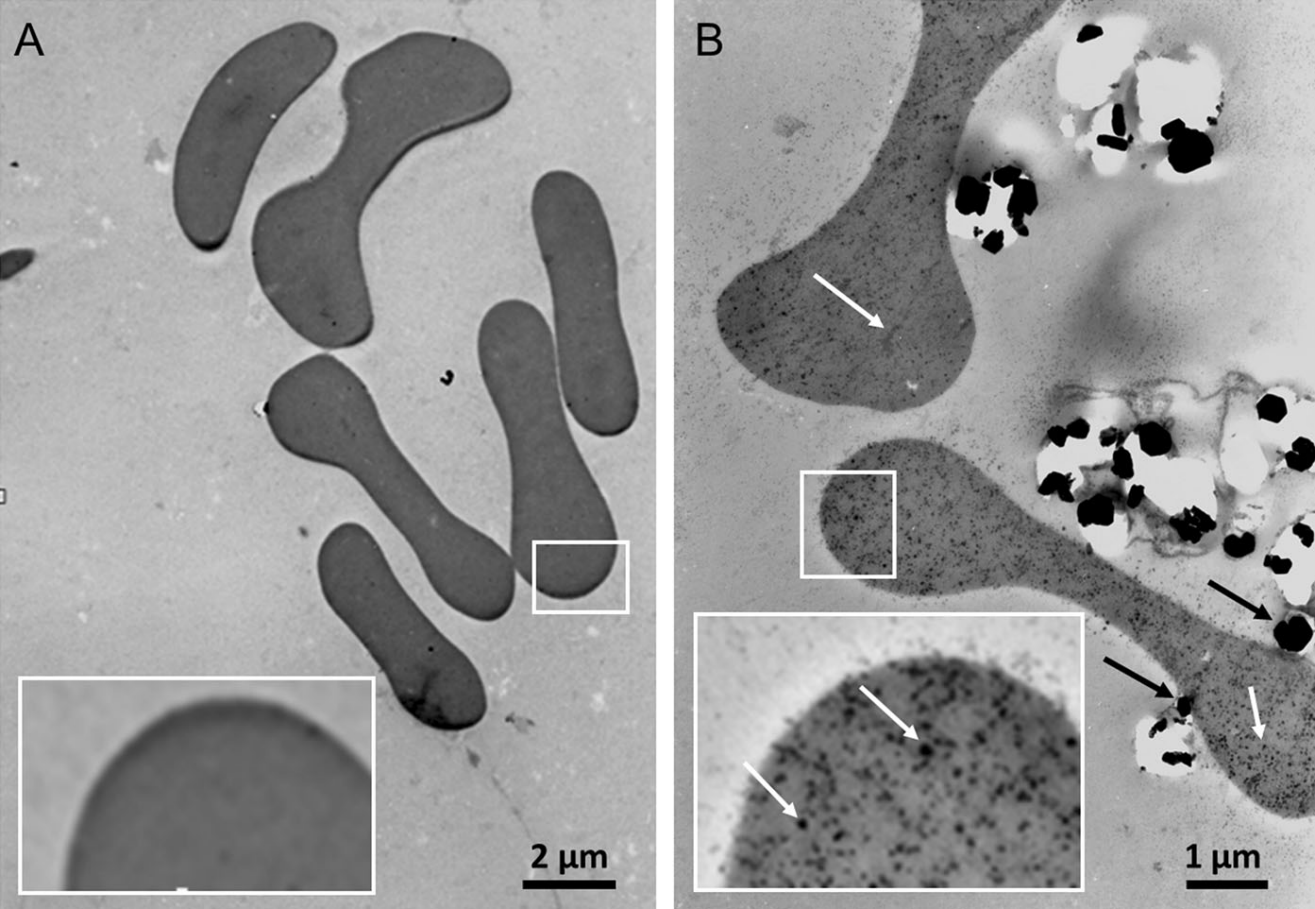
**a** The effect of PBS and **b** GdF_3_:2.5 %Ce^3+^,2.5 %Tb^3+^ (5 mg/mL, 1 h, 37 °C) on human erythrocytes, as observed using a transmission electron microscope. The agglomerates of nanoparticles attached to the erythrocytes membrane (**b**, *black arrows*) and the single nanoparticles inside of erythrocytes (**b**, *white arrows*) were observed

Using a transmission electron microscope (TEM), we were able to observe RBC membrane-bound aggregates of nano-gadoparticles as well as single (non-aggregated) nano-gadoparticles inside of certain RBCs (Fig. 10b). Because RBCs are non-phagocytic cells, our results confirm that nano-gadoparticles can cross their cell membrane barriers. Although the internalization of nanoparticles was observed within human (Rothen-Rutishauser et al. 2006) and mouse erythrocytes, (Soler et al. 2007; Nemmar et al. 2014) the particular mechanism of their entering into these types of cells is still unknown.

Taken together, RBCs exposed to the different concentrations of nanoparticles did not change their shape (Figs. 6, 7) and did not undergo hemolysis, as was the case with the control cells. These results indicate that binding of nanoparticles to the RBC membrane, as observed by SEM and TEM, does not affect the RBC membrane structure and its permeability. Data obtained in our studies demonstrated the lack of toxicity of the novel synthesized nanoparticles on the RBC membrane molecular functionality. However, the binding of nanoparticles to the RBCs does not preclude the presence of some toxic effects, both on the RBC functions as well as their rheology properties.

## Conclusions

A group of nanocrystalline fluorides with multifunctionality in their properties of luminescence and magnetism were synthesized and thoroughly analyzed. The unusual properties observed were consequences of the magnetism of the Gd^3+^-containing matrices and the Ce^3+^, Eu^3+^ and Tb^3+^ doping, which resulted in the intense luminescence. The hydrothermal method of their synthesis was developed and optimized to obtain single-phase products. The morphology of the products obtained was strongly dependent on the host compound used. We observed formation of ring-like structures for GdF_3_-based materials and nanospheres with a diameter of approximately 15 nm for BaGdF_5_-based particles or large spheres with an average size of 150 nm for those based on the NaGdF_4_ compound.

All samples prepared were found to be highly excitable via UV radiation, resulting in green (Tb^3+^) or red (Eu^3+^) luminescence. The highest luminescence was observed for the BaGdF_5_:Ce^3+^,Tb^3+^ sample. Additionally, GdF_3_:Ce^3+^,Tb^3+^ samples exhibited intense emission. The luminescence of Eu^3+^-doped materials was lower than those of Tb^3+^-activated materials, and the highest Eu^3+^ emission was recorded for the BaGdF_5_:Ce^3+^,Eu^3+^ sample.

In conclusion, the investigated nanoparticles exhibit paramagnetism, as well as probable evidence of superparamagnetic behavior at low temperatures. Intraparticle and interparticle interactions are negligible due to the insufficient overlapping of the 4*f* orbitals of the Gd^3+^ ions, and no signs of conduction-electron mediated indirect coupling. Doped lanthanide ions (Ce^3+^, Eu^3+^ and Tb^3+^), which are required from the point of view of luminescent properties, do not significantly influence or even slightly improve the magnetic properties compared to GdF_3_ (Wang et al. 2012). Gd^3+^ ions are decisive for the changes of magnetic behavior, *e.g.*, in comparison with previously studied CeF_3_ or CeF_3_: 20 %Tb^3+^ (Grzyb et al.). Most importantly, nanoparticles preserve their magnetic characteristics when in the form of a suspension in distilled water, which is promising for the possible biological applications.

The cytotoxicity studies against the human erythrocytes indicate that the synthesized nanoparticles are potentially non-toxic, as they did not cause the RBC shape changes nor did they alter their membrane structure and permeabilization. However, binding of nanoparticles to the RBC membrane observed in this study suggests that they may modify the RBC function and properties. Therefore, our findings are crucial for the further applications of BaGdF_5_-, GdF_3_-, or NaGdF_4_-based materials, which are frequently investigated for use in the optoelectronics, phosphors, or biomedical areas.

## Electronic supplementary material

Supplementary material 1 (DOCX 756 kb)
