# Supplementary material for: Synthesis, characterization, and cytotoxicity in human erythrocytes of multifunctional, magnetic, and luminescent nanocrystalline rare earth fluorides
Source: J Nanopart Res. 2015 Oct 5;17(10):399. doi: 10.1007/s11051-015-3191-2 (PMC4594090; doi:10.1007/s11051-015-3191-2)
Supplement: Supplementary file 1 — Supplementary material 1 (DOCX 756 kb) [file 11051_2015_3191_MOESM1_ESM.docx]

**Figure S1.** Temperature dependence of the magnetization M (left scale) and of the reciprocal DC magnetic susceptibility 1/χ (right scale) in GdF_3_:Ce^3+^,Tb^3+^ and NaGdF_4_:Ce^3+^,Tb^3+^. The magnetic mass susceptibilities *χ_g_* were determined at 300 K.

**Figure S2.** Magnetic field dependence of the magnetization M in GdF_3_:Ce^3+^,Tb^3+^ and NaGdF_4_:Ce^3+^,Tb^3+^ powders measured at 2, 10 and 300 K.
